# Mechanical Properties and Microstructures of Solid Waste Composite-Modified Lateritic Clay via NaOH/Na_2_CO_3_ Activation: A Sustainable Recycling Solution of Steel Slag, Fly Ash, and Granulated Blast Furnace Slag

**DOI:** 10.3390/ma18143307

**Published:** 2025-07-14

**Authors:** Wei Qiao, Bing Yue, Zhihua Luo, Shengli Zhu, Lei Li, Heng Yang, Biao Luo

**Affiliations:** 1 School of Engineering and Technology, China University of Geosciences (Beijing), Beijing 100083, China; 3102230001@email.cugb.edu.cn (W.Q.); luozhihua@cugb.edu.cn (Z.L.); 2102240132@email.cugb.edu.cn (S.Z.); 2102220105@email.cugb.edu.cn (H.Y.); 2 Institute of Geology and Geophysics, Chinese Academy of Sciences, Beijing 100029, China; lilei232@mails.ucas.ac.cn; 3 College of Civil Engineering, Hunan University, Changsha 410082, China

**Keywords:** steel slag, fly ash, ground granulated blast furnace slag, soil modification, sodium hydroxide, sodium carbonate

## Abstract

The utilization of steel slag (SS), fly ash (FA), and ground granulated blast furnace slag (GGBFS) as soil additives in construction represents a critical approach to achieving resource recycling of these industrial by-products. This study aims to activate the SS-FA-GGBFS composite with a NaOH solution and Na_2_CO_3_ and employ the activated solid waste blend as an admixture for lateritic clay modification. By varying the concentration of the NaOH solution and the dosage of Na_2_CO_3_ relative to the SS-FA-GGBFS composite, the effects of these parameters on the activation efficiency of the composite as a lateritic clay additive were investigated. Results indicate that the NaOH solution activates the SS-FA-GGBFS composite more effectively than Na_2_CO_3_. The NaOH solution significantly promotes the depolymerization of aluminosilicates in the solid waste materials and the generation of Calcium-Silicate-Hydrate and Calcium-Aluminate-Hydrate gels. In contrast, Na_2_CO_3_ relies on its carbonate ions to react with calcium ions in the materials, forming calcium carbonate precipitates. As a rigid cementing phase, calcium carbonate exhibits a weaker cementing effect on soil compared to Calcium-Silicate-Hydrate and Calcium-Aluminate-Hydrate gels. However, excessive NaOH leads to inefficient dissolution of the solid waste and induces a transformation of hydration products in the modified lateritic clay from Calcium-Silicate-Hydrate and Calcium-Aluminate-Hydrate to Sodium-Silicate-Hydrate and Sodium-Aluminate-Hydrate, which negatively impacts the strength and microstructural compactness of the alkali-activated solid waste composite-modified lateritic clay.

## 1. Introduction

Adding additives to soil aims to improve its mechanical properties, enabling in situ soil to meet engineering and construction requirements. The lateritic clay investigated in this study is distributed in the middle and lower reaches of the Yangtze River. This soil exhibits favorable mechanical properties at low moisture content but becomes highly plastic when the moisture content is higher [1]. Additionally, its strength decreases dramatically with increasing moisture content [2,3]. Due to the climatic conditions in the lateritic clay distribution area [4,5], roadbeds and slopes constructed with lateritic clay often suffer from deformation and erosion [6,7]. Using solid waste as backfill material can promote the sustainable recycling of solid waste [8] and avoid the waste of land resources caused by stacking or landfilling solid waste [9].

Steel slag (SS), fly ash (FA), and ground granulated blast furnace slag (GGBFS) are bulk solid wastes in industrial production. In China, the annual production of industrial solid wastes, represented by SS, FA, and GGBFS, is measured in billions of tons [9]. SS, FA, and GGBFS all exhibit gelling properties [10,11,12] and therefore possess the potential for application in roadbed soil improvement.

Sodium hydroxide and sodium carbonate can activate solid wastes such as steel slag (SS), fly ash (FA), and ground granulated blast furnace slag (GGBFS), enhancing their effectiveness as soil modifiers [13,14,15,16,17]. Existing research indicates that alkalis promote the early hydration of steel slag [18]. However, alkali-activated steel slag exhibits more microcracks and unhydrated particles than hardened cement paste [19]. As silicon-alumina-rich industrial byproducts, FA and GGBFS generate additional hydration products in alkaline environments [20,21], which synergize with SS to improve lateritic clay modification. The free CaO in SS can create an alkaline environment and participate in the formation of a C-S-H gel. Additionally, due to its larger particle size, SS can form a better gradation effect with GGBFS and FA. GGBFS is rich in CaO, SiO_2_, and Al_2_O_3_, while FA is characterized by high silicon and aluminum (SiO_2_ + Al_2_O_3_ > 70%) but low calcium content. When the three are compounded, the high calcium content in SS and GGBFS can supplement the calcium deficiency in FA, promoting the formation of C-S-H gel [22,23].

The dosage of alkaline activators critically influences gel formation [24,25]. When sodium carbonate is used as an activator, its efficacy depends on both dosage and environmental alkalinity [26]. Therefore, optimal NaOH concentration in NaOH-activated composites and appropriate Na_2_CO_3_ dosage in Na_2_CO_3_-activated composites are essential to achieve superior lateritic clay modification.

In this study, an SS-FA-GGBFS composite was prepared using response surface methodology. Unconfined compressive strength (UCS) was adopted as the evaluation criterion for activation efficacy. When preparing lateritic clay specimens modified by NaOH-activated and Na_2_CO_3_-activated SS-FA-GGBFS composites, the water content of the lateritic clay used is 15%, and during the specimen preparation, all specimens are compacted to a compaction degree of 93%. The microstructures of lateritic clay modified by NaOH-activated and Na_2_CO_3_-activated SS-FA-GGBFS composites were characterized via SEM. XRD analysis was performed to identify mineralogical changes in the modified lateritic clay. Based on microscopic and compositional analyses, the effects of NaOH concentration on the performance of NaOH-activated composites and the effects of Na_2_CO_3_ dosage on the performance of Na_2_CO_3_-activated composites were investigated.

## 2. Materials and Methods

### 2.1. Materials

**Figure 1:**
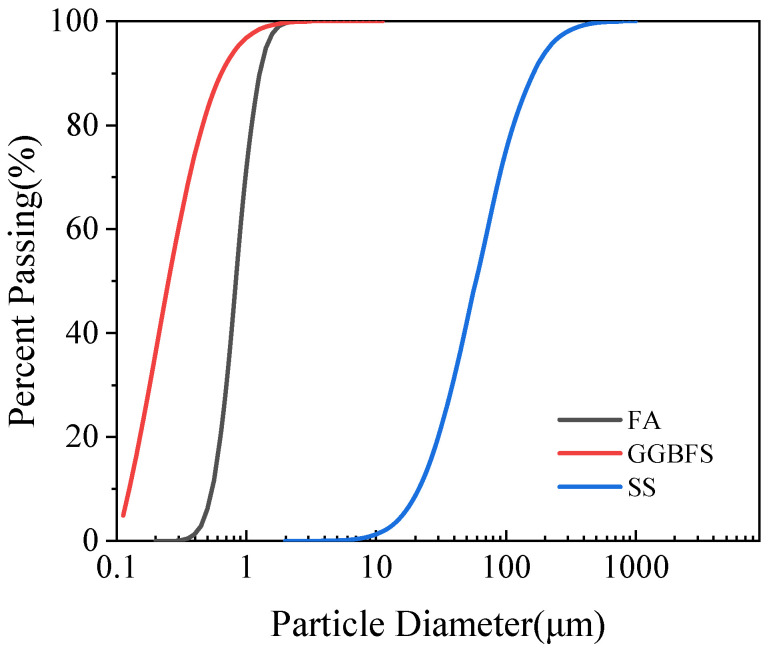
Particle size distributions of SS, FA, and GGBFS.

In this study, SS was sourced from a steel plant in Shijiazhuang City, Hebei Province, China. FA was Class F fly ash (produced by anthracite or bituminous coal combustion with low CaO content) from a thermal power plant in Gongyi City, Henan Province, China. GGBFS was collected from a blast furnace slag powder processing plant in Xinxiang City, Henan Province, China. All three solid waste materials were preprocessed into powder form. The particle size distributions of the three solid waste powders were determined using a wet laser particle size analyzer, and the results are shown in Figure 1.

**Figure 2:**
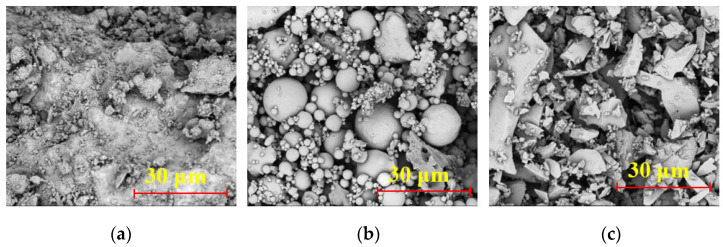
Micro-morphology of: (**a**) SS, (**b**) FA, and (**c**) GGBFS.

The micro-morphology of the three solid waste powders was observed using a scanning electron microscope (Phenom ProX G6, Thermo Fisher, Waltham, MA, USA). The SEM was operated in secondary electron mode at an acceleration voltage of 5 kV. Micrographs of the three solid waste powders are shown in Figure 2.

**Figure 3:**
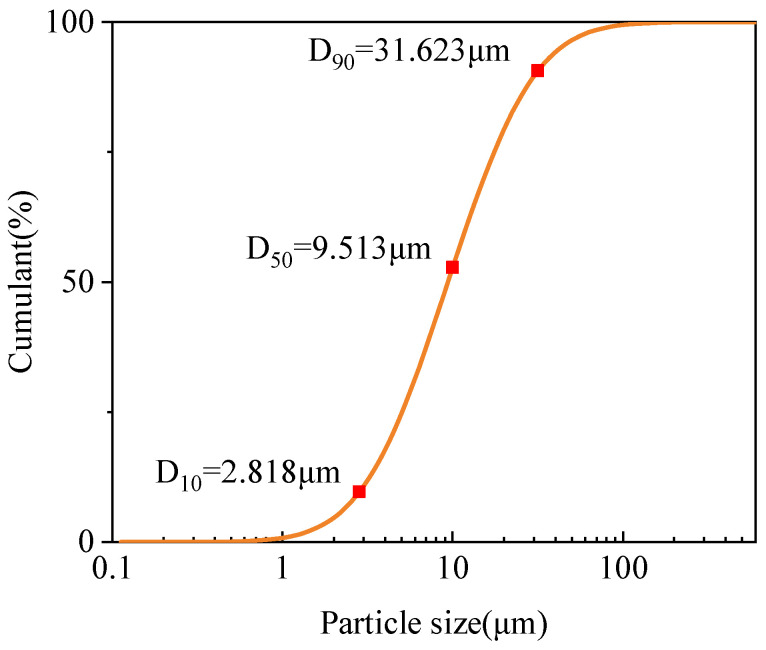
Particle size of lateritic clay after milling.

The lateritic clay reinforced by a solid waste-based cementitious binder with SS-FA-GGBFS as the main components was collected from a newly excavated highway slope in Nanchang City, Jiangxi Province, China. The sampling site has a subtropical monsoon climate with abundant precipitation in spring and summer [27]. To test the water content of lateritic clay under different climatic conditions, samples were collected after 14 consecutive days of no precipitation and 48 consecutive hours of precipitation, respectively. According to the Unified Soil Classification System (USCS) [28], the lateritic clay is classified as clayey silt (CL). The water content and natural density of the in situ lateritic clay were tested. The lateritic clay was dried in an oven at 105 °C until constant weight. The dried lateritic clay lumps were then crushed into particles smaller than 0.6 mm using a crusher, and the ground lateritic clay was used as the raw material for the tests. The particle size test was carried out on the milled lateritic clay, and the test results are shown in Figure 3.

To determine the relative chemical compositions of the three solid wastes, X-ray fluorescence (XRF) analysis was conducted using an X-ray fluorescence spectrometer (Series 2, Bruker, Billerica, USA). The results, expressed in oxide form, are shown in Table 1. The data reveal that SS exhibits high calcium/iron content but relatively low silicon/aluminum content. FA has high silicon/aluminum content but relatively low calcium content; in GGBFS, the contents of CaO, SiO_2_, and Al_2_O_3_ all exceed 10%.

The Atterberg limits of the lateritic clay were determined according to ASTM D4318 [29]. The results indicate that the liquid limit is 47.7%, the plastic limit is 18.1%, and the plasticity index is 29.6%. To investigate the water content variability under different climatic conditions, two soil samples were collected from the same site during the rainy season (June) and dry season (October), and their water contents were measured. The results show that the water contents of the lateritic clay after 14 days without rainfall and 48 h of continuous rainfall are 11.63% and 24.53%, respectively. The natural density of the soil is 1.63 g/cm^3^. Unconfined compressive strength (UCS) tests were performed on the rainy and dry season samples via uniaxial compression. The UCS values of the lateritic clay in the rainy and dry seasons were 116 kPa and 34 kPa, respectively. The increase in water content caused by rainfall led to a reduction in the UCS exceeding 70%.

### 2.2. Specimen Preparation

Before sample preparation, a mixture of SS, FA, and GGBFS was first prepared. This SS-FA-GGBFS mixture was then mixed with a NaOH solution or Na_2_CO_3_. Finally, the NaOH-activated or Na_2_CO_3_-activated SS-FA-GGBFS composite was combined with lateritic clay. The resulting NaOH-SS-FA-GGBFS-lateritic clay or Na_2_CO_3_-SS-FA-GGBFS-lateritic clay mixture was placed in a cylindrical mold and compacted using the static pressure method.

Considering the climatic conditions of the lateritic clay’s location, a water content of 15% is close to and slightly higher than the natural water content of the lateritic clay during most periods. Selecting this water content facilitates the application of research results to actual engineering projects in the region. Meanwhile, a 15% water content is also slightly lower than the optimum water content and plastic limit of the lateritic clay, which is beneficial for specimen molding.

The specific specimen preparation steps are as follows: First, water was uniformly sprayed onto the lateritic clay using a spray bottle until the moisture content reached 15%. The moistened lateritic clay was then sealed and allowed to equilibrate for 12 h. Based on the optimal mix proportions determined from previous response surface methodology tests, SS, FA, and GGBFS were mixed at a ratio of 1.7:1.3:1 to form the SS-FA-GGBFS composite. The NaOH, with a specific concentration, and Na_2_CO_3_, with a specific dosage, need to be mixed with the SS-FA-GGBFS composite prior to mixing the SS-FA-GGBFS composite with the lateritic clay. The mass ratio of the NaOH solution to the solid waste composite is 0.38. After the mixing of the lateritic clay and solid waste composite is complete, the lateritic clay–solid waste composite will be used to make specimens for stress testing. The specimens for the pressure test are cylindrical, which has a diameter of 39 mm and a height of 80 mm, in accordance with ASTM D4832 [30]. Table 2 presents the preparation schemes for SS-FA-GGBFS-modified lateritic clay specimens using NaOH solutions of different concentrations and Na_2_CO_3_ at different dosages as activators. When using the NaOH-activated SS-FA-GGBFS or Na_2_CO_3_-activated SS-FA-GGBFS, the amount of the activated SS-FA-GGBFS added was determined according to the standard, where the ratio of the dry mass of SS-FA-GGBFS in the activated SS-FA-GGBFS material to the dry mass of lateritic clay was 0.22.

For each mix proportioning scheme, the NaOH-activated SS-FA-GGBFS-modified lateritic clay and the Na_2_CO_3_-activated SS-FA-GGBFS-modified lateritic clay were cured for three curing ages: 3 days, 7 days, and 21 days. All specimens were cured at a temperature of 20 °C ± 2 °C. During specimen preparation, the compaction degree of each specimen was controlled at 93%.

The strength loss of lateritic clay at higher moisture contents is more pronounced, so investigating the strength performance of modified lateritic clay under higher moisture contents is of greater potential value for practical engineering applications. However, lateritic clay with a high moisture content exhibits perfect plasticity, which is not conducive to specimen preparation (e.g., molding and demolding). Thus, the moisture content of the lateritic clay for specimen preparation was set at 15% lower than the plastic limit, and a simulated precipitation procedure was conducted after curing the modified lateritic clay to increase its moisture content. During titration, the burette dripping rate was 0.5 mL/min, and water was dropped onto the specimen‘s upper surface. At this rate, the precipitation amount per unit area of the specimen exceeded the lower limit of heavy precipitation defined by meteorology (16 mm/h).

**Figure 4:**
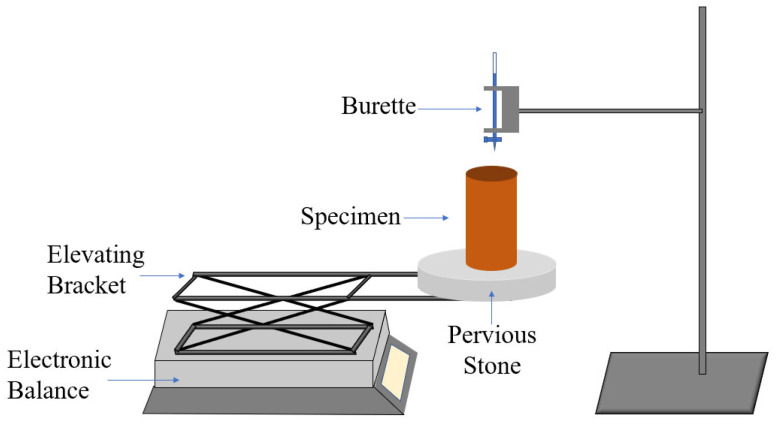
Device for rainfall simulation on specimens.

After the specimens are cured, they are taken out of the curing chamber and placed on the titration device, as shown in Figure 4. Water is dripped onto the top of the specimens with a burette at a rate of 0.5 mL/min until the mass of the specimens increases by 10%.

### 2.3. Test Methods

**Figure 5:**
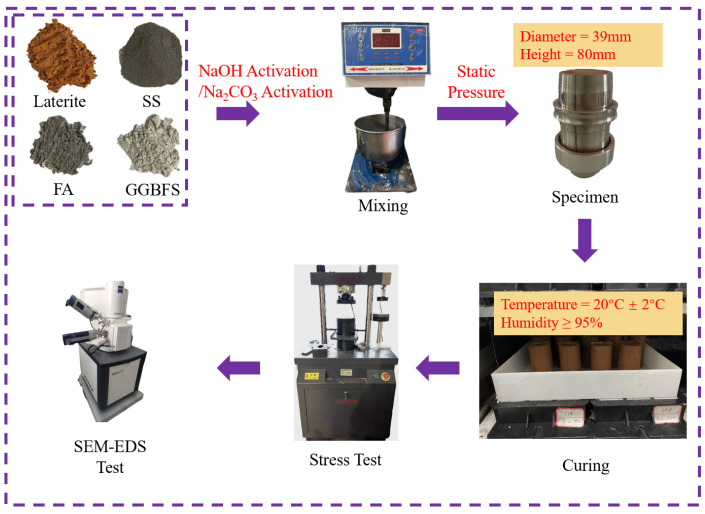
Specimen preparation and testing procedures.

Uniaxial compression tests were conducted on lateritic clay modified by activated SS-FA-GGBFS composites. Using unconfined compressive strength (UCS) as an indicator, the effects of varying NaOH concentrations in NaOH-activated SS-FA-GGBFS and different Na_2_CO_3_ contents in Na_2_CO_3_-activated SS-FA-GGBFS on lateritic clay modification were investigated. The uniaxial test was conducted using a 10 kN displacement-controlled compression module in a universal testing machine at a compression speed of 2 mm/min. Subsequently, SEM observations were conducted on the NaOH-activated SS-FA-GGBFS-modified lateritic clay specimens and Na_2_CO_3_-activated SS-FA-GGBFS-modified lateritic clay specimens to explore new structures formed in the modified lateritic clay. Energy-dispersive X-ray spectroscopy (EDS) was used to analyze the elemental composition of hydration products in both types of modified lateritic clay specimens. Figure 5 is a schematic diagram of the entire experimental process.

## 3. Results and Analysis

### UCS of NaOH-Activated SS-FA-GGBFS-Modified Lateritic Clay Specimens and Na_2_CO_3_-Activated SS-FA-GGBFS-Modified Lateritic Clay Specimens

**Figure 6:**
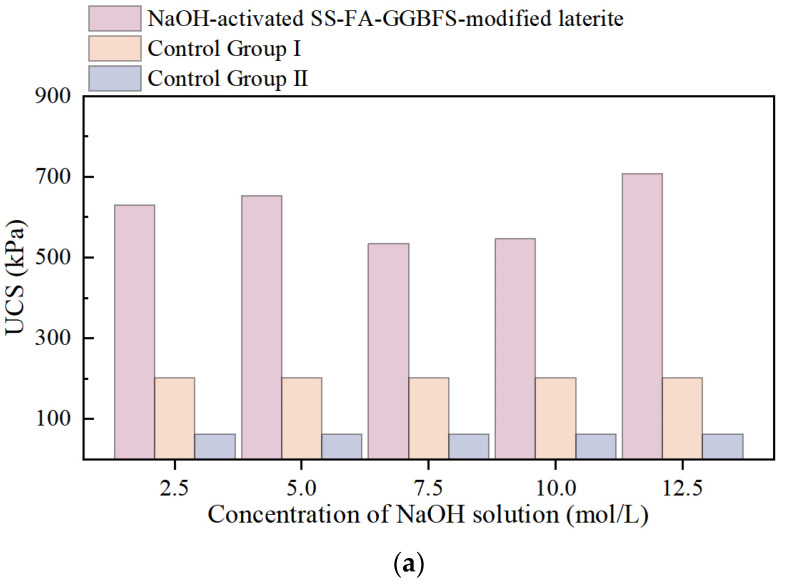

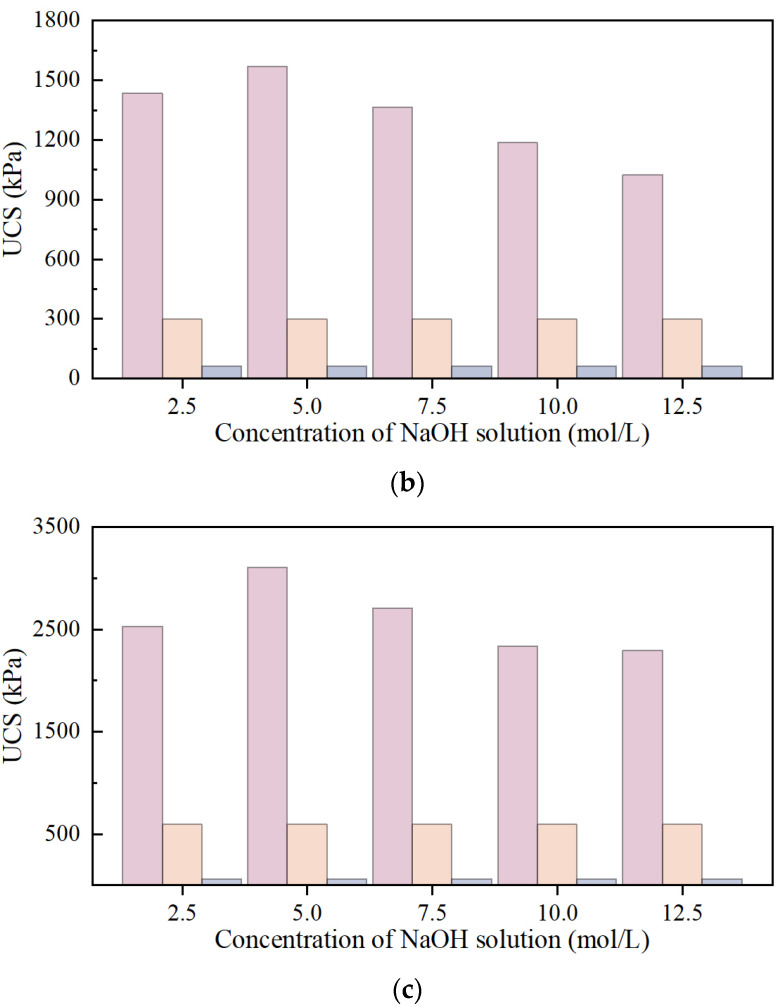
Comparison of uniaxial compression test results of lateritic clay modified by NaOH-activated SS-FA-GGBFS with curing times of (**a**) 3 days, (**b**) 7 days, and (**c**) 21 days.

Figure 6 shows the results of single-axis compression tests on NaOH-activated SS-FA-GGBFS-modified lateritic clay after 3 days, 7 days, and 21 days. As a control, Figure 5 also presents the SS-FA-GGBFS-modified lateritic clay (Control Group I) and the unmodified lateritic clay (Control Group II) under the same curing ages.

The unconfined compressive strength (UCS) of NaOH-activated SS-FA-GGBFS-modified lateritic clay at curing ages of 3 days, 7 days, and 21 days significantly increased compared with that of SS-FA-GGBFS-modified lateritic clay. Additionally, the strength of NaOH-activated SS-FA-GGBFS-modified lateritic clay continuously improved with the increase in curing age. When comparing the influence of the NaOH solution concentration on the strength of NaOH-activated SS-FA-GGBFS-modified lateritic clay, it was found that the strength of the modified lateritic clay generally first increased and then decreased with the increase in NaOH concentration. This indicates that there is an optimal concentration for NaOH as an activator. Based on the experimental results, the suitable concentration of NaOH was determined to be around 5 mol/L. The compressive strength of the 5 mol/L NaOH-activated SS-FA-GGBFS-modified lateritic clay cured for 3 days increased by 944% compared with the unmodified lateritic clay. The compressive strength of the specimen cured for 7 days increased by 2140%, and the compressive strength of the specimen cured for 21 days increased by 4860%.

**Figure 7:**
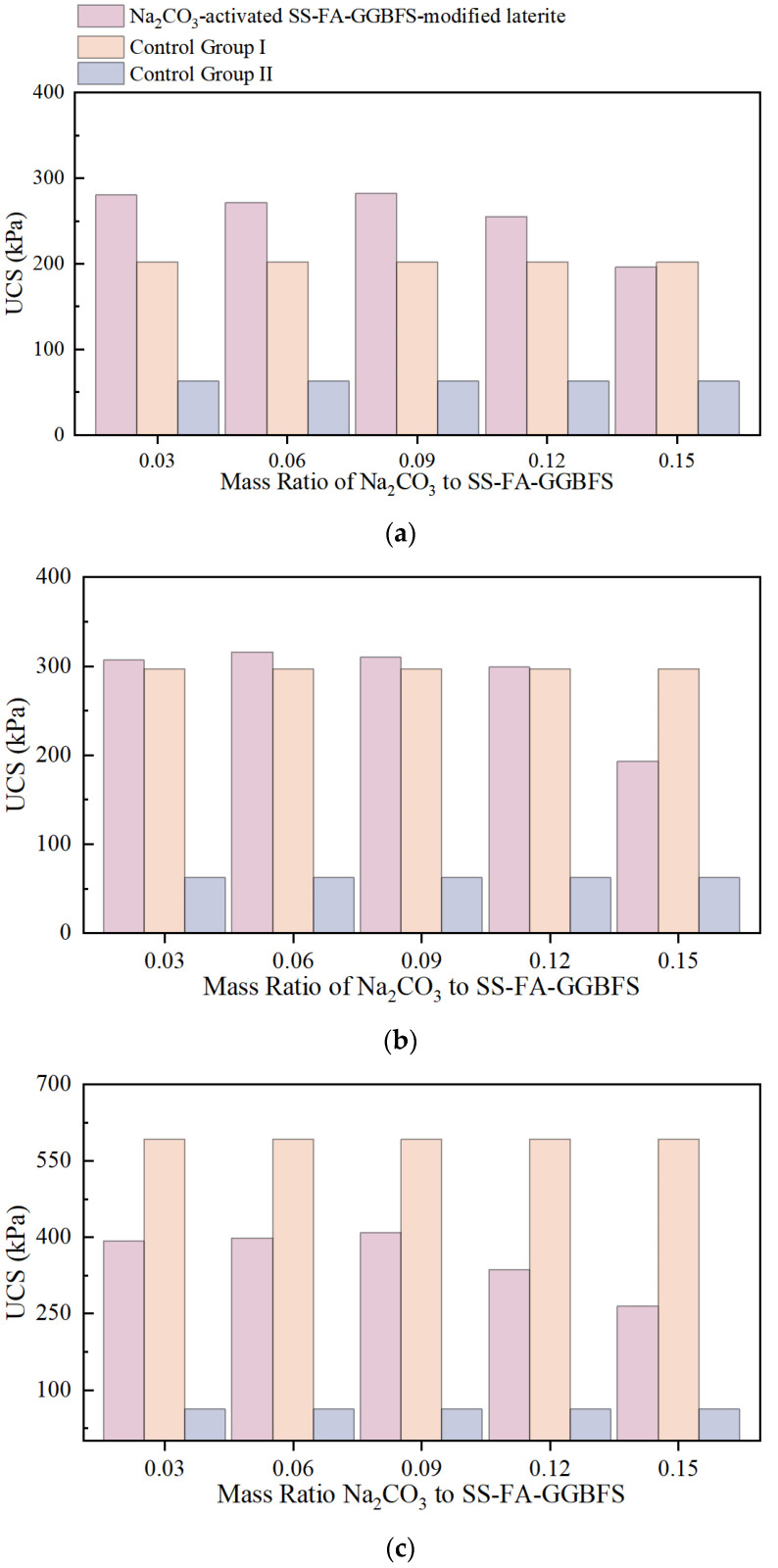
Comparison of uniaxial compression test results of lateritic clay modified by Na_2_CO_3_-activated SS-FA-GGBFS with curing times of (**a**) 3 days, (**b**) 7 days, and (**c**) 21 days.

Figure 7 shows the results of single-axis compression tests on Na_2_CO_3_-activated SS-FA-GGBFS-modified lateritic clay after 3 days, 7 days, and 21 days.

For Na_2_CO_3_-activated SS-FA-GGBFS-modified lateritic clay with a curing age of 3 days, when the mass ratio of Na_2_CO_3_ to SS-FA-GGBFS was less than 0.12, the unconfined compressive strength (UCS) of the modified lateritic clay showed a certain degree of improvement compared with that of SS-FA-GGBFS-modified lateritic clay. When the mass ratio was 0.09, the UCS of Na_2_CO_3_-activated SS-FA-GGBFS-modified lateritic clay increased by approximately 40% compared with SS-FA-GGBFS-modified lateritic clay.

For Na_2_CO_3_-activated SS-FA-GGBFS-modified lateritic clay with a curing age of 7 days, when the mass ratio of Na_2_CO_3_ to SS-FA-GGBFS was below 0.09, the strength of the modified lateritic clay increased slightly (by less than 10%). However, when the mass ratio reached 0.15, the strength of the modified lateritic clay significantly decreased.

For Na_2_CO_3_-activated SS-FA-GGBFS-modified lateritic clay with a curing age of 21 days, the UCS of all modified lateritic clay samples was lower than that of SS-FA-GGBFS-modified lateritic clay.

Following uniaxial compression, SS-FA-GGBFS-modified lateritic clay specimens activated by different activators exhibit distinct failure behaviors. For specimens activated with NaOH, those cured for 3 days primarily undergo brittle splitting failure, whereas specimens cured for 7 days or longer transition to brittle tensile failure. In contrast, all specimens activated with Na_2_CO_3_ exhibit brittle splitting failure, accompanied by lateral bulging deformation.

These results indicate that Na_2_CO_3_-activated SS-FA-GGBFS-modified lateritic clay exhibits faster early-stage strength development compared with SS-FA-GGBFS-modified lateritic clay, but its late-stage strength growth is limited. Consequently, adding Na_2_CO_3_ as an activator to SS-FA-GGBFS performed worse in modifying lateritic clay than using SS-FA-GGBFS alone without activation.

It should be noted that when the NaOH-activated SS-FA-GGBFS is added to the lateritic clay, the water in the NaOH solution may change the original moisture content of the soil, thereby affecting its strength. In fact, if all the water in the NaOH solution penetrates into the particles of the lateritic clay, the initial moisture content of the NaOH-activated SS-FA-GGBFS-modified lateritic clay would be about 8% higher than that of the Na_2_CO_3_-activated SS-FA-GGBFS-modified lateritic clay. However, due to the rapid hydration of the SS-FA-GGBFS mixture in the early stage, which consumes part of the water in the NaOH solution, the solvent water brought by the NaOH solution will not be fully absorbed by the lateritic clay. If the same initial water content is to be achieved for the SS-FA-GGBFS-modified lateritic clay activated by both activators, it is necessary to reduce the initial water content of the NaOH-activated variant during preparation. In practice, however, this exact value is difficult to determine because of the early hydration of SS-FA-GGBFS. Thus, when comparing the strengths of the two modified lateritic clays (NaOH-activated vs. Na_2_CO_3_-activated), it is crucial to note that their moisture contents inherently differ.

## 4. Microstructural and Elemental Characterization

### 4.1. Comparative Analysis of Microstructure

**Figure 8:**
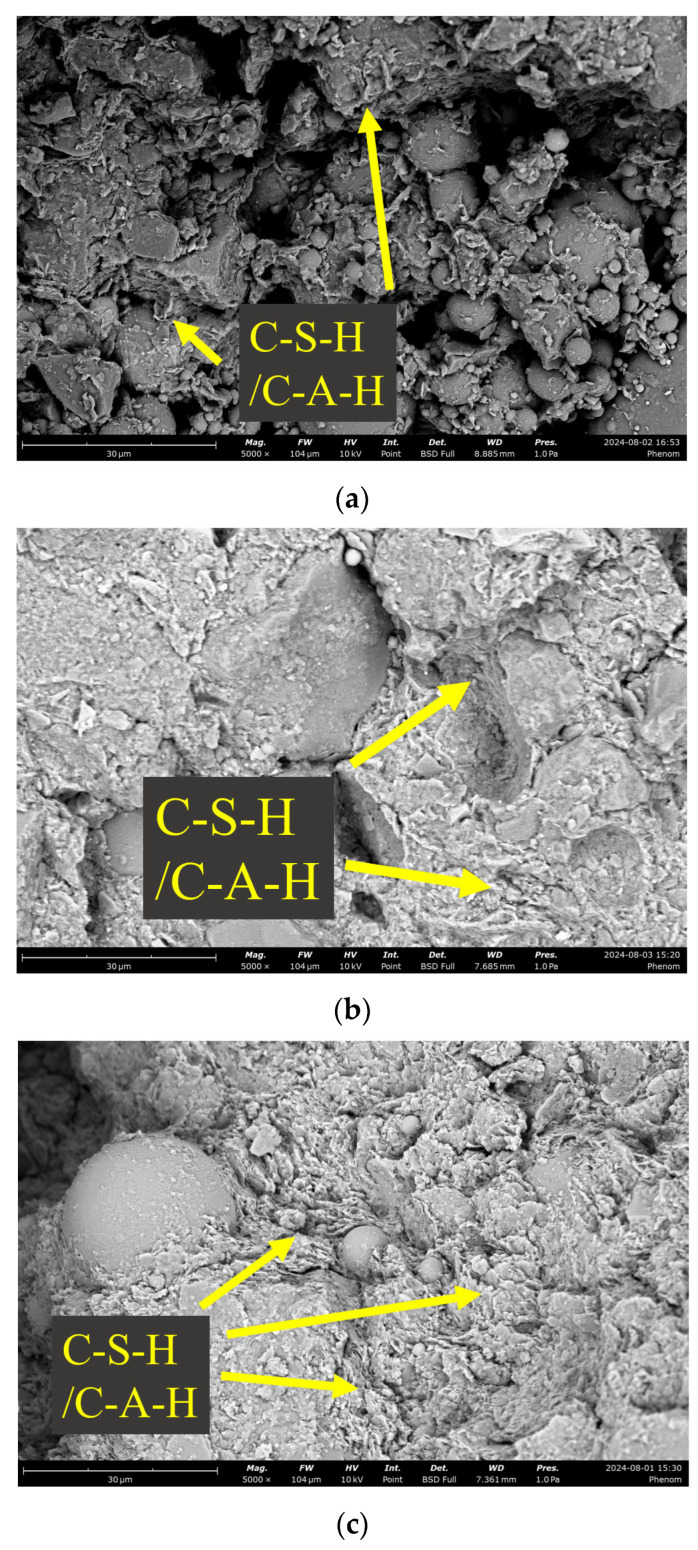
SEM images of NaOH-activated SS-FA-GGBFS-modified lateritic clay with curing times of (**a**) 3 days, (**b**) 7 days, and (**c**) 21 days.

Figure 8 presents the SEM images of NaOH-activated SS-FA-GGBFS-modified lateritic clay with a NaOH solution concentration of 5 mol/L after 3 days, 7 days, and 21 days of curing.

By comparing the scanning electron microscopy (SEM) images of NaOH-activated SS-FA-GGBFS-modified lateritic clay at different curing ages, it is clearly evident that the microstructure of the modified lateritic clay becomes denser as the curing age increases. As shown in Figure 8a, in the SEM image of NaOH-activated SS-FA-GGBFS-modified lateritic clay after 3 days of curing, the contours of soil particles are very clear, and the morphologies of GGBFS particles and FA particles show no obvious changes. Moreover, the area of unfilled pores accounts for over 30% of the soil volume, with pore sizes concentrated between 5 and 20 μm.

As depicted in Figure 8b, in the SEM image after 7 days of curing, a certain amount of flocculent hydration products can be observed on the surface of soil particles. These flocculent products fill the voids in the soil and bond the soil particles together. At this stage, part of the GGBFS particle surface is bonded by flocculent hydration products, but some outer surfaces of GGBFS remain relatively smooth with limited attachment of hydration products. Meanwhile, the number of unfilled pores in the modified lateritic clay has been significantly reduced, with pore diameters not exceeding 10 μm.

As illustrated in Figure 8c, in the SEM image after 21 days of curing, the number and area of pores are significantly reduced, and the solid waste particles and soil particles are bonded into a whole by a large amount of hydration products. Moreover, the pores between soil particles are completely filled, leaving only a few isolated micropores within the hydration products, with pore sizes not exceeding 5 μm.

Through comparative analysis of scanning electron microscopy (SEM) images of Na_2_CO_3_-activated SS-FA-GGBFS-modified lateritic soil at different curing ages, it was observed that the proportion of internal cracks in the modified soil exhibited a gradual increase with extended curing periods.

**Figure 9:**
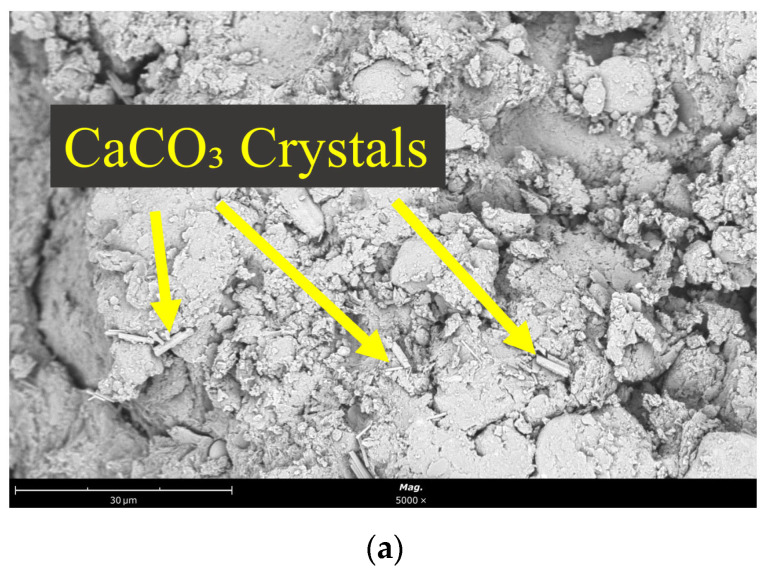

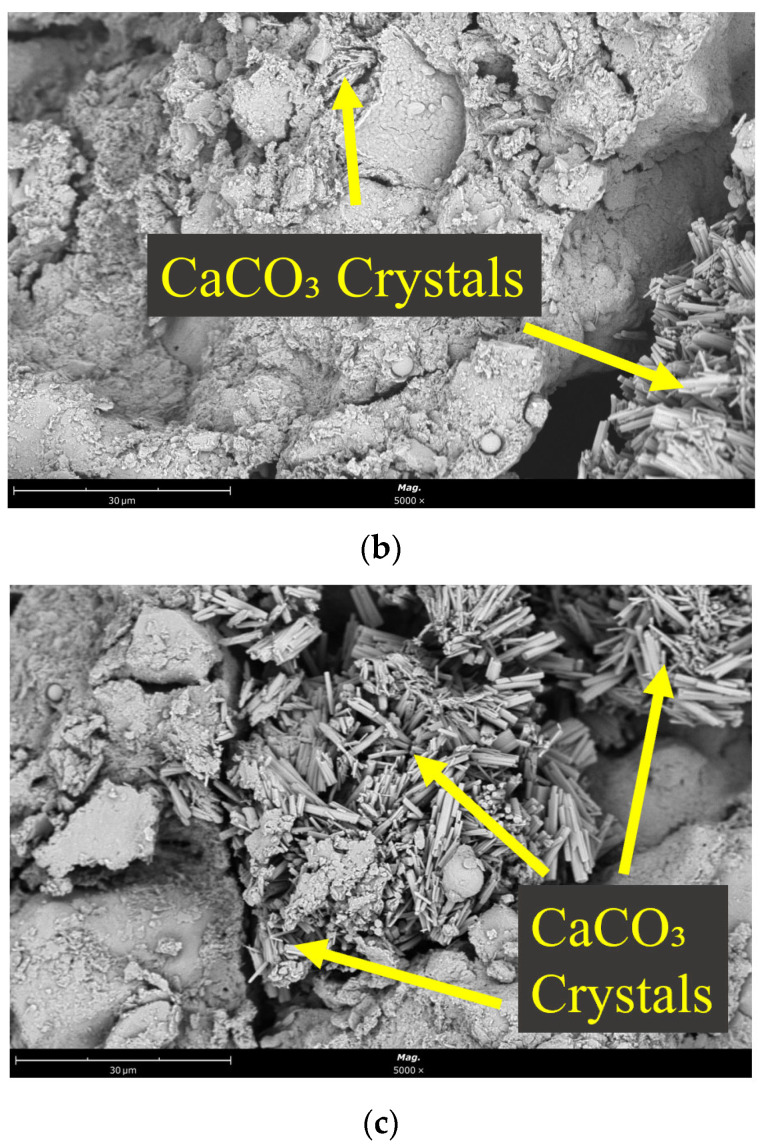
SEM images of Na_2_CO_3_-activated SS-FA-GGBFS-modified lateritic clay with different curing times of (**a**) 3 days, (**b**) 7 days, and (**c**) 21 days.

At the 3-day curing stage (Figure 9a), sparse calcium carbonate (CaCO_3_) crystals in isolated single-crystal morphology were found filling the pores between soil particles and solid waste aggregates. These crystalline structures remained relatively discrete, showing limited interconnection with the surrounding matrix.

Notably, by the 7-day curing period (Figure 9b), a significant increase in CaCO_3_ crystal content was evident, with the formation of clustered crystalline aggregates. These clusters displayed enhanced intergrowth but simultaneously introduced distinct cracks perpendicular to the crystal growth direction at the interface between soil particles and crystalline phases. This morphological transition indicated the onset of mechanical stress induced by crystalline expansion.

Further progression to the 21-day curing stage (Figure 9c) revealed a continued increase in CaCO_3_ crystal density, accompanied by a shift from unidirectional to radial growth patterns. The crystalline clusters exhibited pronounced detachment from adjacent soil particles, with clear separation at the interface boundaries. This structural evolution suggested that crystal growth dynamics had transcended the pore-filling stage, leading to matrix disruption.

Mechanistically, the early-stage formation of CaCO_3_ crystals (3 days) contributed to pore filling and densification of the modified soil matrix, thereby enhancing its early-stage strength through improved particle interlocking. However, prolonged curing induced excessive crystalline growth, generating mechanical stresses that disrupted the cohesive structure of the soil-solid waste composite. The directional expansion forces exerted by growing crystals led to particle separation at the interface, compromising the material integrity and ultimately exerting a negative impact on the long-term mechanical properties of the modified lateritic clay.

Overall, from the micro-morphology presented in the scanning electron microscope (SEM) images, it can be seen that under the same curing age conditions, the hydration product structure generated in the NaOH-activated SS-FA-GGBFS-modified lateritic clay is more abundant than that in the Na_2_CO_3_-activated SS-FA-GGBFS-modified lateritic clay. With the increase in time, the integrity and compactness of the NaOH-activated SS-FA-GGBFS-modified lateritic clay structure continue to increase, while the integrity of the Na_2_CO_3_-activated SS-FA-GGBFS-modified lateritic clay gradually deteriorates with the increase in curing age. This explains why the unconfined compressive strength of the NaOH-activated SS-FA-GGBFS-modified lateritic clay continuously improves with the increase in curing age, whereas that of the Na_2_CO_3_-activated SS-FA-GGBFS-modified lateritic clay shows a decline. The reason for the strength decline in the Na_2_CO_3_-activated SS-FA-GGBFS-modified lateritic clay is that Na_2_CO_3_ reacts with the free calcium oxide in SS-FA-GGBFS to produce calcium carbonate crystals, and the mechanical stress from crystal growth leads to the destruction of the overall structure of the Na_2_CO_3_-activated SS-FA-GGBFS-modified lateritic clay.

### 4.2. Elemental Characterization

**Figure 10:**
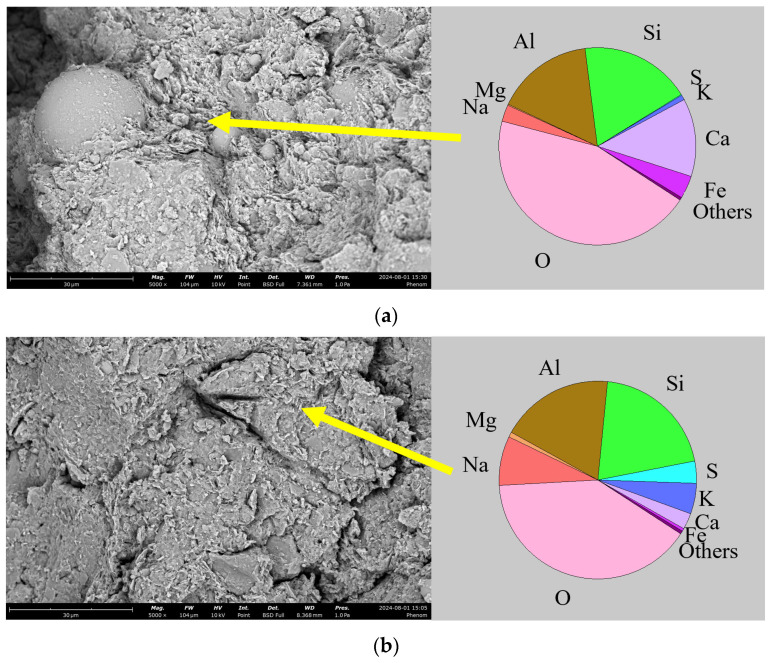
SEM images and EDS analysis of (**a**) NaOH-activated SS-FA-GGBFS-modified lateritic clay treated with 5 mol/L NaOH solution and (**b**) NaOH-activated SS-FA-GGBFS-modified lateritic clay treated with 12.5 mol/L NaOH solution.

Figure 10 presents the X-ray diffraction patterns of two samples: the NaOH-activated SS-FA-GGBFS-modified lateritic clay treated with a 5 mol/L NaOH solution and cured for 21 days, and that treated with a 12.5 mol/L NaOH solution and cured for 21 days. Meanwhile, Figure 10 also presents the atomic percentage of different chemical elements obtained by EDS analysis of hydration products in the two samples.

**Figure 11:**
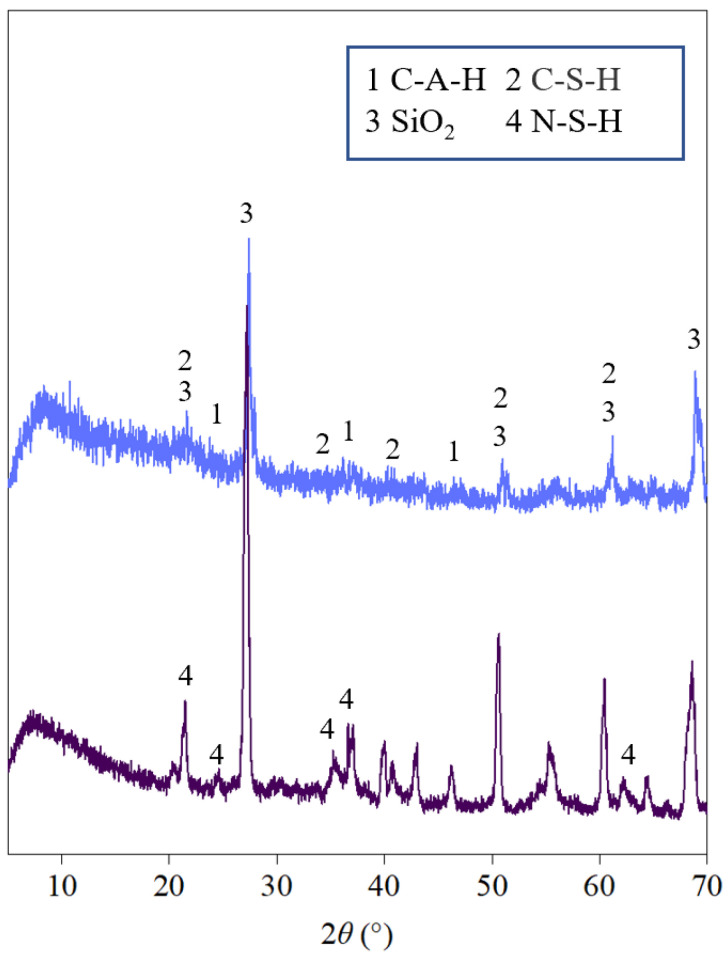
The XRD patterns of 5 mol/L NaOH-activated SS-FA-GGBFS-modified lateritic clay and 12.5 mol/L NaOH-activated SS-FA-GGBFS-modified lateritic clay.

Through comparison, it was found that the hydration products in the SS-FA-GGBFS-modified lateritic clay activated with 5 mol/L NaOH are denser than those in the SS-FA-GGBFS-modified lateritic clay activated with 12.5 mol/L NaOH. In the 5 mol/L NaOH-activated SS-FA-GGBFS-modified lateritic clay, the hydration products exist in a gel-like form, filling the pores between soil particles and solid waste particles and wrapping the surfaces of most soil particles. In contrast, the hydration products in the 12.5 mol/L NaOH-activated SS-FA-GGBFS-modified lateritic clay exhibit a fish-scale-like morphology, adhering to the surfaces of soil particles with a relatively thinner hydration product layer. The EDS test results show that the enrichment degree of calcium in the hydration products of the 5 mol/L NaOH-activated SS-FA-GGBFS-modified lateritic clay is higher than that in the 12.5 mol/L NaOH-activated counterpart, while the sodium content in the hydration products of the 12.5 mol/L NaOH-activated SS-FA-GGBFS-modified lateritic clay is higher than that in the 5 mol/L NaOH-activated sample. The XRD patterns obtained from the X-ray diffraction tests conducted on 5 mol/L NaOH-activated SS-FA-GGBFS-modified lateritic clay and 12.5 mol/L NaOH-activated SS-FA-GGBFS-modified lateritic clay are shown in Figure 11.

The XRD patterns indicate that the hydration products in the 5 mol/L NaOH-activated SS-FA-GGBFS-modified lateritic clay are mostly calcium silicate and calcium aluminate in an amorphous state, whereas those in the 12.5 mol/L NaOH-activated SS-FA-GGBFS-modified lateritic clay are sodium silicate, and the sodium silicate exhibits a higher degree of crystallinity.

### 4.3. Modification Mechanisms Solid Waste Composite and Alkali-Activated Solid Waste Composite Modified Lateritic Clay

When NaOH and Na_2_CO_3_ are used as activators for SS-FA-GGBFS, an appropriate concentration of NaOH solution (5 mol/L) enables rapid dissolution of the glassy phase on the surface of SS-FA-GGBFS, primarily composed of silicon and aluminum oxides. During this process, calcium, aluminum, and silicon are released and dissolved into the solvent water of the solution and the interparticle water within the lateritic clay matrix, thereby promoting the formation of C-S-H (Calcium-Silicate-Hydrate) and C-A-H (Calcium-Aluminate-Hydrate) gels. These gels serve as cohesive matrices bridging solid waste particles and soil aggregates, thereby augmenting the compressive strength of the modified lateritic clay. With prolonged curing, the continuous polymerization of these gels ensures sustained mechanical enhancement. Specifically, C-S-H (Calcium-Silicate-Hydrate) and C-A-H (Calcium-Aluminate-Hydrate) gels consume substantial interparticle water in the lateritic clay. Concurrently, after this water is consumed, the dense gels fill and seal the pores between clay particles, preventing external water infiltration. This dual mechanism—water consumption and pore sealing—significantly enhances the water-exposed strength of NaOH-activated SS-FA-GGBFS-modified lateritic clay.

However, excessively high NaOH concentrations (e.g., 12.5 mol/L) trigger rapid nucleation and deposition of hydration products on particle interfaces, thereby hindering the ongoing polymerization reactions of aluminosilicates [31,32]. An excessively alkaline environment inhibits the dissolution of calcium oxide from SS-FA-GGBFS and preferentially promotes the formation of sodium-based hydration products, such as N-S-H (Sodium-Silicate-Hydrate) and N-A-H (Sodium-Aluminate-Hydrate) gels [33]. Compared to the calcium-based C-S-H and C-A-H gels, the N-S-H and N-A-H gels exhibit a less dense microstructure and provide an inferior contribution to mechanical strength development due to weaker interparticle bonding [34]. As demonstrated in Figure 7b, excessive NaOH concentrations are directly associated with a thinner and less continuous hydration product layer, characterized by reduced coverage of soil particle surfaces and increased porosity.

In contrast to NaOH activation, the mechanism governing Na_2_CO_3_ activation of SS-FA-GGBFS composite involves distinct physicochemical pathways. The lower alkalinity of Na_2_CO_3_ compared to NaOH results in significantly reduced dissolution of the glassy phases in SS-FA-GGBFS, primarily composed of calcium aluminosilicates. However, when Na_2_CO_3_ dissolves in the interparticle water within the lateritic clay matrix, it hydrolyzes to form a weakly alkaline environment, prompting the active components in SS, FA, and GGBFS to depolymerize and release Ca^2+^, Si^4+^, and Al^3+^ ions. The CO_3_^2−^ dissociated from sodium carbonate directly increases the CO_3_^2−^ concentration in the solution. The combination of CO_3_^2−^ from Na_2_CO_3_ and free Ca^2+^ ions released by solid waste depolymerization induces ion supersaturation in the system, driving the crystallization of CaCO_3_. These crystals initially exhibit a rhombohedral morphology and fill the interparticle pores, contributing to early-age (3-day) strength enhancement through mechanical interlocking and reduced void ratio. However, the cementitious efficacy of CaCO_3_ differs fundamentally from that of C-S-H/C-A-H gels. As a rigid crystalline phase, CaCO_3_ lacks the colloidal plasticity of amorphous gels, leading to weaker interfacial bonding with soil particles. Unlike the C-S-H and C-A-H colloids, CaCO_3_ crystals cannot form a continuous and dense water-insulating structure in the lateritic clay matrix. Consequently, external water can still easily infiltrate between the particles of the Na_2_CO_3_-activated SS-FA-GGBFS-modified lateritic clay. This induces water absorption and particle separation via electrostatic interactions, thereby compromising the integrity of the soil mass. Moreover, more critically, prolonged curing (≥7 days) triggers uncontrolled CaCO_3_ crystal growth, which follows a radial pattern and generates significant crystallographic stresses within the matrix. These stresses induce microcracking at the crystal–soil particle interface, as confirmed by SEM images showing distinct cleavage planes and detachment zones. The resulting structural discontinuities degrade the load-transfer capacity, culminating in progressive strength deterioration.

## 5. Conclusions

This study conducted unconfined compression tests on lateritic clay modified by NaOH-activated SS-FA-GGBFS and Na_2_CO_3_-activated SS-FA-GGBFS. The results showed that when NaOH was used as the activator, there existed an optimal concentration range (5 mol/L) for NaOH-activated SS-FA-GGBFS to achieve the maximum compressive strength of modified lateritic clay. In contrast, Na_2_CO_3_-activated SS-FA-GGBFS-modified lateritic clay exhibited better early strength development, but its strength decreased with the increase in curing age. To analyze the modification mechanisms of NaOH-activated and Na_2_CO_3_-activated SS-FA-GGBFS in lateritic clay, SEM and EDS were used to conduct microstructural observation and elemental analysis of the modified lateritic clay. The main conclusions of this study are summarized as follows:

(1) The strength of NaOH-activated SS-FA-GGBFS-modified lateritic clay generally increases with curing age. For NaOH concentrations ranging from 2.5 to 12.5 mol/L, the strength first increases and then decreases with concentration, peaking at 5 mol/L. After 7+ days of curing, the 5 mol/L modified clay shows a strength several dozen times higher than unmodified clay under rainfall-induced water content changes, meeting the requirement for maintaining strength in precipitation.
(2) The strength of lateritic clay modified by Na_2_CO_3_-activated SS-FA-GGBFS generally decreases with the increase in curing age. When the mass ratio of Na_2_CO_3_ to SS-FA-GGBFS ranges from 0.03 to 0.09, Na_2_CO_3_ can enhance the strength of the modified lateritic clay to a certain extent at the curing age of 3 days. However, with the extension of curing time, Na_2_CO_3_ has a negative effect on the strength of the modified lateritic clay.
(3) The NaOH solution can dissolve the dense glassy phase on the surface of SS-FA-GGBFS, which is predominantly composed of silicon and aluminum oxides, thereby accelerating the dissolution and release of elements such as calcium, aluminum, and silicon from SS-FA-GGBFS.
(4) Due to its weaker alkalinity, Na_2_CO_3_ has a less effective dissolution effect on the glassy phase of the SS-FA-GGBFS surface compared to NaOH. It primarily relies on the pore-filling effect of calcium carbonate crystals formed by the reaction between carbonate ions and free calcium oxide in SS-FA-GGBFS to improve the strength of the modified soil. However, over time, the continuous growth of calcium carbonate crystals generates mechanical stresses that disrupt the integrity of the modified soil, thereby reducing its strength.
(5) The addition of NaOH solution accelerates the reaction of SS-FA-GGBFS blends, promoting the formation of C-S-H and C-A-H gels, which are primarily responsible for the strength enhancement of modified lateritic clay. However, an optimal NaOH concentration is critical; too high a concentration of NaOH solution impedes the dissolution efficiency of solid waste precursors and induces a phase transition from calcium-based (C-S-H/C-A-H) to sodium-based (N-S-H/N-A-H) hydration products.

## Figures and Tables

**Table 1:** Chemical composition of lateritic clay and three kinds of solid wastes.

Oxides	Composition (wt.%)			
	Lateritic Clay	SS	FA	GGBS
Calcium oxide	0.74	41.22	3.60	59.31
Silicon dioxide	52.99	6.32	35.71	16.31
Aluminum oxide	18.78	2.88	37.34	10.24
Ferric oxide	13.48	22.44	9.86	1.46
Magnesium oxide	1.04	5.68	0.46	5.63
Sulphate oxide	0	0.59	-	-
Potassium oxide	5.06	0.33	1.66	0.57
Manganese oxide	0.17	3.80	0.09	1.00
LOI (Loss on ignition)	4.97	12.77	2.05	-

**Table 2:** Mix proportioning scheme of activator and SS-FA-GGBFS.

Proportioning Scheme	Type of Activator	Activator Form	Solution Concentration (mol/L)	Mass of Activator Added per 100 g SS-FA-GGBFS (g)
1	NaOH	Solution	2.5	38
2			5	
3			7.5	
4			10	
5			12.5	
6	Na_2_CO_3_	Solid	-	3
7				6
8				9
9				12
10				15

## Data Availability

The raw data supporting the conclusions of this article will be made available by the authors on request.
